# Supplementary material for: Are carbon emissions trading and green financial instruments synergistic? -Comprehensive quantitative research based on content analysis
Source: PLoS One. 2024 Mar 7;19(3):e0298601. doi: 10.1371/journal.pone.0298601 (PMC10919601; doi:10.1371/journal.pone.0298601)
Supplement: S1 Data — (ZIP) [file pone.0298601.s001.zip › Data/地级市Z_F工作报告.zip!/╦─┤¿/╫╩╤⌠╩╨/└┤╫╘╣1⁄2╓┌║┼í╛┬φ┐╦╩2╛▌═°í┐_2016.docx]

各位代表：

　　现在，我代表市人民政府向大会报告工作，请予审查，并请市政协委员和其他列席人员提出意见。

　　“十二五”时期经济社会发展回顾

　　过去五年，市政府在省委、省政府和市委的坚强领导下，在市人大、市政协的监督支持下，与全市人民一道，团结拼搏、开拓创新，较好完成了“十二五”规划确定的目标任务。

　　经过五年奋斗，经济平稳较快增长，产业结构持续优化。经济实力迈上新台阶，全市地区生产总值年均增长11.9%、快于全省1.1个百分点，2012年上升到全省第10位，2013年迈上千亿平台，2015年是2010年的1.9倍；地方一般公共预算收入是2010年的2.5倍，实现了经济发展量质双增。结构优化取得新成效，产业结构由23：53：24优化为19.8：55.3：24.9；非公有制经济占比由54.7%提升至59.2%。产业培育取得新突破，四川现代、唯品会等重大项目竣工投产，造车、食品、医药、电子商务等产业在全省优势明显，特色效益农业规模不断壮大。

　　经过五年奋斗，基础建设强力突破，城乡面貌明显变化。城镇建设快速推进，中心城区主要干道基本形成，九曲河综合整治成效明显，天府新区资阳片区建设全面推进，全市城镇建成区面积达到237平方公里，城镇化率年均提高1.4个百分点。园区发展能力明显增强，形成了79平方公里的6大产业园区，工业集中度达75.5%，园区生产、就业、服务、纳税能力持续提升。交通建设快速突破，成都天府国际机场落户资阳；成渝客专通车运营，资阳迈入高铁时代；遂资眉等3条高速公路建成通车，实现县县通高速。水利建设取得积极进展，旱区群众期盼半个世纪的毗河供水一期工程全线加快建设；五年新增有效灌溉面积18万亩，解决了148.6万人饮水安全问题。能源通信设施加快建设，电网建设取得明显成效，500千伏变电站建成投用，天然气开发和综合利用步伐加快，建成全光网城市。

　　经过五年奋斗，改革开放成效显著，发展活力持续释放。各项改革全面推进，行政审批制度改革持续推进；国家政策性减税50.7亿元，其中“营改增”减税1.6亿元；农业农村、医疗卫生、户籍制度等改革取得积极进展。开放合作全面拓展，五年引进到位国内省外资金1767亿元、外资4.3亿美元，全市“三类500强企业”达41户、投资合作项目64个，引进金融保险机构9户，与52个省级部门、高校等建立战略合作关系。

　　经过五年奋斗，社会事业持续发展，人民生活明显改善。财政民生支出575.3亿元，是“十一五”的2.6倍；城乡居民收入年均分别增长12.2%、14.2%；新增城镇就业12.75万人；社会保障体系不断健全，企业退休人员基本养老金提高478元；社会救助水平持续提升，城乡低保月人均补助分别提高57.5%、87%；教育、卫生、文化、体育等事业全面进步；生态文明建设不断加强，成功创建全国双拥模范城市和省级环保模范城市、省级环境优美示范城市；依法治市深入推进，社会大局和谐稳定。

　　2015年工作总结

　　2015年，经济下行压力之大前所未有、发展改革稳定任务之重前所未有、矛盾风险挑战之多前所未有，市政府在省委、省政府和市委的坚强领导下，在市人大、市政协的监督支持下，牢牢把握稳中求进工作总基调，扎实推进稳增长促改革调结构惠民生防风险各项工作，较好完成了市三届人大五次会议确定的目标任务。实现地区生产总值1270.4亿元、增长8.8%，增速居全省第7位，地方一般公共预算收入61.8亿元、增长10.8%，城乡居民人均可支配收入分别增长8.3%、10.1%。

　　一年来，主要做了以下工作：

　　一、聚力推动发展，经济平稳较快增长

　　抓政策落实稳定增长。坚持把稳增长摆在突出位置，认真贯彻落实国省稳增长系列政策，出台工业、要素保障等5个方面23条稳增长政策措施，制定化解涉农企业资金困难实施方案，推动实体经济稳增长。投资稳步增长，海大轮胎等74个重点项目竣工投用，字库山公园二期等187个重点项目加快推进，全社会固定资产投资实现1045.7亿元、增长15.1%。工业平稳发展，造车、食品、医药等优势产业不断壮大，规模以上工业增加值增长9.8%。服务业加快发展，增加值增长10%，占地区生产总值比重提高1.1个百分点，社会消费品零售总额实现464.7亿元、增长14.1%，增速保持全省第一。农业提质发展，增加值增长3.9%，粮食产量“九连增”，水果、蔬菜等特色农业达208万亩。非公有制经济健康发展，增加值实现751.5亿元、增长9.3%，对经济增长贡献率达62.4%。

　　抓工作谋划理清思路。编制“六大规划”，引领支撑未来发展。编制“十三五”规划，明确未来五年发展思路、定位、目标和重点工作；修编资阳城市总体规划，建设宜居宜业城市；编制航空都市规划，培育“一核两极多点”市域城镇群；编制临空经济区规划，引导高端制造业、现代服务业集聚发展；编制中韩（资阳）产业园规划，深化与韩国在汽车制造、食品医药等领域的交流合作；修编土地利用总体规划，推进城乡存量建设用地挖潜利用和高效配置。在深入研判形势和分析市情的基础上，进一步明确了“抓加快发展稳增长、抓薄弱环节补短板、抓矛盾化解防风险、抓自身建设带队伍”的工作思路，实施一系列工作方案和行动计划，推动经济平稳运行。

　　抓突出矛盾解决难题。坚持问题导向，集中梳理重点项目推进中的192个问题、工业企业运行中的140个问题、服务业企业发展中的39个问题，逐一研究解决。破解资金难题，开展银企对接40余次，新增贷款72.7亿元；协调金融机构以展期、延期等方式直接转贷近20亿元；建立应急转贷金2.35亿元，帮助企业转贷10.7亿元；新增3000万元充实市级政策性担保公司资本金，帮助166户企业担保融资10亿元。加强税收征管，实现地方税收收入45.9亿元；加大争取力度，到位上级财政转移支付141.2亿元、债券资金82.3亿元。强化用地保障，实施农村土地整理、城乡建设用地增减挂钩等项目，争取建设用地指标2044.5公顷；加强已供未用土地、闲置土地清理，强化已批未征土地征收，盘活存量土地。

　　抓企业帮扶共度难关。扎实开展“走企业、解难题”活动，建立完善市级领导联系企业、企业家接待日等制度，推动工业企业产值增幅超过20%的173户企业挖潜增效，54户产值负增长企业止滑扭亏，7户停产半停产企业恢复生产。全面落实楼市新政，出台公积金调整政策、购房奖励等9条措施，举办房交会，刺激住房消费，房地产销售面积452.5万平方米、增长6.1%。

抓创新创业驱动发展。大力推进“大众创业、万众创新”，落实国、省部署要求，出台支持农民工返乡创业等23条措施，资阳经济开发区和简阳、安岳工业集中发展区被评为省新型工业化产业示范基地，创新创业孵化器加快建设。加强科技项目支撑引领，出台支持中小企业科技创新等21条政策措施；政府、企业科技投入分别达1.7亿元、3.8亿元；实施国省高新技术项目42项，高新技术企业达41户，高新技术产业化效益居全省第4位。企业上市实现零突破，国光农化在深交所挂牌。加强商标品牌建设，新创驰名商标1件、省著名商标4件、四川名牌9个。

二、强力夯实基础，发展条件明显改善

　　城乡建设步伐加快。坚持“改造完善提升”思路，围绕完善城市功能、提升城市品质落实工作举措，在一些领域取得了新进展。规划编制有序开展，《资阳城市总体规划》已通过省上复审，中心城区总体城市规划设计、沱江资阳城区段综合治理总体规划设计加快推进，凤岭公园等7个重点项目规划编制形成初步成果，城市夜景照明、农贸市场等专项规划编制完成。城市建设有力推进，完成投资108亿元、增长21.3%，城镇化率达到39.5%、提高1.3个百分点。中心城区车城大道延长线改造等18个项目竣工投用，滨江路堤景观带等33个项目加快建设、现代大道北延线等32个项目前期工作扎实推进；策划准备了中心城区干道提升等5个方面重点项目，为实现三年城市品质明显提升打下了良好基础；4个县城和发展新区建设取得明显成效。天府新区资阳片区加快建设，路网框架成型，主题功能突出，产业项目集聚，完成投资22亿元，马鞍山观景台一期等6个项目建成和部分投用，世茂产业生态城等22个项目加快推进，丹景生态移民家园五期等7个项目开工建设。村镇建设扎实推进，14个“百镇建设行动”试点镇完成投资11.8亿元，建成幸福美丽新村300个、新村聚居点330个。

　　城市环境明显改善。围绕“畅洁绿美、规范有序”要求，逐步理顺城管体制，市区联动、部门协同、市民参与，大力推进中心城区市容市貌十大专项治理行动，基本实现一年明显变化目标。大力整治农贸市场，困扰市民十余年的鱼市口、皇龙、刘家湾3个马路市场被彻底取缔，后西街等6个农贸市场完成改造整治，天王农产品批发市场一期、孙家坝临时农贸市场建成投用，农贸市场整治取得阶段性成效；大力整治环境卫生，清除了城区干道和城中村、城郊结合部暴露垃圾和卫生死角垃圾，实施主次干道机扫作业，城区环境卫生明显改善；大力整治户外广告，强力拆除违规设置、污损破旧广告，城区广告管理逐步规范；大力整治客运交通秩序，严查严处影响畅通、危及安全、酒后驾车等交通违法行为，有序取缔人力三轮车营运，禁止电动三轮车违法上路行驶，城区交通及客运秩序明显好转。整治占道经营、城区扬尘污染、噪音污染、防违治违以及市政基础设施建设、美化亮化等工作取得积极进展。

　　基础建设扎实有效。综合交通加快建设，完成投资141.3亿元、增长5.9%。成都天府国际机场保障基地加快建设，机场岩土工程试验段开工建设，成渝客专、成都二绕资阳段建成通车，成都经济区环线高速南段加快建设，资潼广高速、机场高速、资三快速通道等项目前期工作有序推进，完成国省干线大中修268公里，新改建县乡道239公里、村道354公里。水利建设力度加大，完成投资53.3亿元、增长35%。毗河供水一期工程全线加快推进，完成投资15亿元，二期工程纳入部省规划；中小水利工程加快建设，解决51.4万农村人口饮水安全。能源通信设施不断完善，建成输变电站7座，城网农网改造加快推进；安岳气田勘探开发成效明显，安岳区块油气处理厂建成投产；4G网络基站达到2000个，农村乡镇4G覆盖率达到75%。

　　生态环境建设力度加大。重拳整治环境污染，扎实推进畜禽养殖、大气、水污染防治工作，畜禽养殖污染治理成效得到国家环保部充分肯定。生态建设不断加强，新营造林25.5万亩，新增森林面积9.8万亩，森林覆盖率达38%，省级园林城市创建经省政府验收。国土资源有效保护，新增耕地2万亩，确保了40.6万公顷耕地保有量、33.8万公顷基本农田面积不减、质量不降。

　　三、着力改革开放，发展活力持续增强

　　各项改革推进有力。简政放权力度加大，政府机构改革顺利完成，非行政许可事项全部取消，政府权力清单初步建立，政府向社会力量购买服务稳妥推进。投融资体制改革加快推进，基金、债券融资力度加大，简阳农商银行正式挂牌。农村综合改革成效明显，土地经营权确权登记基本完成，土地流转收益保证贷款试点等顺利实施。医药卫生体制改革全面深化，基本医疗保障制度和分级诊疗制度全面实施，县级公立医院全部取消药品加成。商事制度改革持续推进，完成工商登记“三证合一”，实现“一照一码”。不动产统一登记制度等改革有序推进。

　　对外开放不断扩大。投资促进成效明显，参加和举办中外知名企业四川行等省市11项投资促进活动，新签约美国安博物流2个世界500强项目，引进到位国内省外资金375.3亿元、外资1.2亿美元。对外贸易稳步发展，进出口总额实现3.4亿美元，新增进出口获权企业21户。交流合作不断拓展，出台融入“一带一路”战略实施方案，着力构建对外开放新格局；与成都、重庆交流合作不断拓展；与南开大学等9个单位签署战略合作协议。

　　四、努力补齐短板，社会民生不断进步

　　民生工程深入推进。省市民生工程、民生实事全面完成，财政民生性支出145.2亿元，占地方公共财政支出比重达68%。精准扶贫有力推进，圆满完成省下47个贫困村、7万人的脱贫任务。就业形势持续稳定，城镇新增就业2.9万人，登记失业率3.97%。社会保障力度加大，全面实行养老、工伤、生育保险市级统筹，城乡居民大病保险启动实施，社会救助能力和精准水平不断提高。住房保障得到加强，保障性安居工程开工11322套、79.2万平方米，竣工9351套、48.4万平方米，完成农村危旧房改造7820户。

　　社会事业协调发展。理清社会事业发展思路，谋划推动一批教育、卫生、文化、体育重点项目和重点工作，为实现社会事业三年增量提质奠定基础。教育事业加快发展，制定实施学前教育三年行动计划、解决中小学大班额实施方案，新开工建设公办幼儿园18所，改造农村校舍、辅助用房等13万平方米，高考本科上线率居全省前列，职业学校学生就业率达98%，成都体育学院整体迁建项目加快推进。医疗服务水平稳步提升，制定实施城区医疗服务能力提升三年行动计划，推进与省内外知名医院合作，支持社会办医和中医药事业发展，完成“三中心”合一，基本公共卫生服务持续加强，单独二孩政策稳妥实施。文化事业不断进步，图书馆、文化馆、农家（社区）书屋等公共文化设施免费开放，建成文艺家之家活动阵地；群众文化活动蓬勃开展，成功举办资阳市第三届社会文化展演。体育设施建设力度加大，群众性体育设施不断完善，资阳游泳馆基本建成。外事侨台、统计、档案、地方志、保密、民族宗教、红十字、老龄、残疾人、妇女儿童等工作取得新成绩。

　　五、全力防范风险，社会保持和谐稳定

　　经济风险总体可控。实体经济风险得到有效管控，帮助企业协调资金、开拓市场、解决问题，没有出现企业大规模停产半停产和大量职工下岗失业现象；一企一策制定化解农业产业化龙头企业信用风险方案，并组织实施。成安渝高速公路复工取得实质性进展，已终止原投资人特许经营权，正重新招标确定新投资人。城建领域遗留问题处置力度加大，大力实施政府性安置房建设三年攻坚行动，在建509万平方米、竣工170万平方米；对城区18个毛地拍卖项目逐一研究处置方案，正按计划积极稳妥推进；排查整治7个问题楼盘，城建领域信访问题下降40%。政府性债务管理更加规范，加强政府性债务管控，健全借管用还良性机制，采取债券置换等方式偿还政府债务111.9亿元，其中市本级18.6亿元。金融风险管控加强，积极化解企业信用风险，规范民间借贷活动，严厉打击非法集资、恶意逃废银行债务等行为，大力清收化解不良贷款，提振了金融机构支持发展的信心。

社会治理不断加强。依法治市深入推进，“六五”普法通过验收，法治建设取得积极进展。平安建设扎实有效，严厉打击各类违法犯罪活动，社会治安形势持续好转；坚持依法治访，解决了一批信访突出问题。安全形势比较平稳，市政府应急指挥平台建成投用，未发生食品药品责任安全事故和重特大安全生产事故。国防动员不断加强，征兵任务圆满完成，民兵预备役应急应战能力提升，安岳、乐至建成省级双拥模范县。

　　六、致力提升效能，自身建设得到加强

　　扎实开展“三严三实”专题教育，深入改进作风，加快转变政府职能，服务发展能力和水平不断提升。加强法治政府建设，自觉接受人大、政协监督，人大代表建议和政协提案办理满意和基本满意率100%。坚持常务会会前学法，严格依法决策，政府信息公开和行政权力网上运行力度加大。加强服务政府建设，共办理73.4万件行政审批和公共服务事项，现场办结率、按时办结率达100%，群众满意率99.96%。加强效能政府建设，健全市政府会议决策、财政资金管理、目标绩效管理等制度，政府机关运行规范高效；深入推进作风建设，大力整治执行不力、推诿扯皮、不愿担当、效率质量不高和不按规则办事等问题。加强廉洁政府建设，严格落实中央“八项规定”、省市“十项规定”精神，深入落实党风廉政建设“两个责任”，监察审计切实加强，反腐倡廉工作扎实推进。

　　各位代表，过去一年的成绩来之不易，经验弥足珍贵。这得益于省委、省政府和市委的坚强领导，得益于市人大、市政协的监督支持和各民主党派、工商联、无党派人士、各人民团体的积极参与，得益于各级各部门和全市人民的艰苦努力。在此，我代表市人民政府，向各位代表、政协委员，向全市广大干部群众、政法干警、驻资部队官兵、民兵预备役人员，向所有关心支持我市发展的各界人士，表示衷心的感谢，并致以崇高的敬意!

　　在总结成绩的同时，我们也清醒地看到，发展中还面临不少困难和问题：经济下行压力仍然很大，地区生产总值、财政收入、城乡居民收入增速放缓。实体经济面临较多困难，工业品出厂价格指数处于近年低点，市场需求不足，产成品库存增加，部分行业、企业产能释放不足；非正常生产企业资源盘活难度大。金融形势不容乐观，新增贷款增速下降，融资难、融资贵仍然存在；企业应收账款增加，部分企业资金紧张，民间借贷潜在风险较大。投资保持高位增长压力较大，企业家信心和投资能力下降，新开工重点项目不足，续建项目剩余投资量减少。新旧增长动力转换亟待提速，科技引领支撑不足，缺乏创新型龙头企业，新增长动力处于培育阶段。发展中的短板明显，城市规划建设管理水平不高，社会事业发展滞后，扶贫开发、环境治理任务艰巨。政府作风建设仍需加强，工作效率、质量和服务水平有待进一步提升。这些问题，我们将高度重视，在今后工作中努力加以解决。

　　“十三五”的总体思路和主要目标

　　未来五年，资阳处于实现追赶跨越的战略机遇期、经济质量效益同步提升的转型发展期和与全国全省同步全面建成小康社会的决战决胜期。我们将全面贯彻党的十八大，十八届三中、四中、五中全会和习近平总书记系列重要讲话精神，深入落实省委、省政府和市委各项决策部署，统筹推进“四个全面”战略布局，切实贯彻五大发展理念，主动适应经济发展新常态，突出创新驱动、投资拉动、产业带动，统筹推进经济建设、政治建设、文化建设、社会建设和生态文明建设，加快建设“三区一都市”，奋力打造全省经济强市，确保与全国全省同步全面建成小康社会。主要预期目标是：主要经济指标保持高于全省平均水平的合理增长，地区生产总值年均增长8%以上，2020年经济总量冲刺2000亿元；人均地区生产总值力争达到和超过全省平均水平。科技创新能力显著增强，产业转型升级取得实效，先进制造业和现代服务业加快发展；人民生活水平和质量明显提高，贫困人口全部脱贫；生态环境质量明显改善；社会事业快速发展；社会文明程度普遍提升。

　　“十三五”时期，我们将着力完成四大重点任务：

　　一是提升产业发展层次，加快构建现代产业体系。突出发展先进制造业，强力打造先进交通装备产业集群，大力培育发展生物医药、电子信息、节能环保、智能装备、天然气等新兴成长型产业。集聚发展现代服务业，大力发展以航空服务、空港物流、货物与服务贸易等为重点的临空产业链和产业集群，促进电子商务、现代物流、生态旅游、康体养生等现代服务业迅速发展。加快推进农业现代化，切实转变农业发展方式，发展适度规模经营，着力构建配套合理、功能完善的现代农业体系。

　　二是推动统筹协调发展，着力形成均衡发展格局。统筹城乡协调发展，坚持以人的城镇化为核心，着力建设活力、美丽、生态、文明的现代化城市。推进城乡良性互动、协调发展。构建现代基础设施体系，加快建设成渝次级综合交通枢纽，加强水利、信息、能源基础设施建设，增强生产生活保障能力。加快建设生态文明新家园，全面推进生态资阳建设，强化环境保护治理，推进资源节约集约循环利用。

　　三是全面深化改革开放，不断释放经济发展活力。全力推进改革攻坚，进一步简政放权，提高政府效能。深化金融改革，着力构建服务实体经济的金融体系。建立完善全面规范、公开透明的预算制度，积极推广政府与社会资本合作模式。着力扩大对外开放，打造国家级内陆航空港发展示范区，参与争创国家综合保税区和内陆自贸区。创新招商引资，着力引进一批龙头型、基地型重点企业和项目。深化多层次区域合作，推进天府新区、空港经济区和总部经济发展等跨区域合作项目建设，协同推进成资一体化发展，扩大渝资合作领域，高水平建设中韩创新创业产业化基地、军民融合产业园和国际旅游文化功能区。

　　四是持续加强社会建设，增进民生福祉促和谐。坚决打赢脱贫攻坚战，实现“四年集中攻坚、两年巩固提升”总体目标，确保贫困村贫困人口全部脱贫。大力发展社会事业，加大财政民生投入，抓好民生工程和民生实事，加快建成覆盖城乡全体居民的社会保障体系，坚持教育优先发展，提升医疗服务质量水平，繁荣发展文化事业。深入推进依法治市，构建办事依法、遇事找法、解决问题用法、化解矛盾靠法的法治良序，确保社会安全稳定。

　　2016年工作安排

　　2016年仍是比较困难的一年，经济工作压力和风险还在加大，我国经济发展不可能通过短期刺激实现V型反弹，可能会经历一个L型增长阶段，需要沉着应对，做好打稳增长持久战的思想准备。我们也看到做好经济工作有很多有利条件：中央实施更加积极的财政政策和稳健的货币政策，加强供给侧结构性改革，配套推进“五大政策”，深入实施“一带一路”、长江经济带战略，加大精准扶贫、西部大开发力度；全省实施“三大发展战略”和“五个统筹”，加快天府新区、成都天府国际机场、中韩创新创业产业化基地建设，规划建设临空经济区；我市经过多年加快发展、夯基蓄势，为发展提供了基础条件。我们将坚定稳增长的信心和决心，以良好的精神状态，全力推进全市经济平稳健康发展。

今年政府工作的总体要求是：全面贯彻落实中央、省委省政府和市委系列重大决策部署，牢固树立五大发展理念，始终保持专注发展、转型发展定力，坚持稳中求进工作总基调，把抓加快发展稳增长作为主旋律，把抓矛盾化解防风险作为关键点，把抓薄弱环节补短板作为突破口，把抓自身建设带队伍作为保障力，努力实现“十三五”发展良好开局。

　　今年主要预期目标建议为：地区生产总值增长8%，地方一般公共预算收入同口径增长9%，城乡居民人均可支配收入分别增长8%、9%，城镇化率提高1.2个百分点。目标建议是按照高于全国、快于全省发展速度提出的，总体上积极可行，但也需要加倍努力才能实现。

　　一、全力稳增长调结构，保持经济平稳发展

　　把稳增长摆在更加突出的位置，紧紧扭住投资拉动、产业支撑、创新驱动，推动产业优化升级，促进经济稳定增长。

　　坚持项目带动投资拉动。强力推进“项目年”活动，发挥投资对经济增长的关键性作用，全社会固定资产投资增长11%。推动项目稳定投资，推进255个市级重点项目建设，确保117个项目按期开工，四川现代汽车配套零部件生产基地等90个项目加快推进，百威罐装生产线等48个项目竣工投用。加强储备争取项目，建立重大项目三级储备管理体系，主动对接国省重大支持政策，全力争取项目和资金。完善机制促进项目，构建“一门受理、转告相关、限时办结”的联动机制，切实提高行政审批效能；强化“六个对接”，多渠道解决资金、土地等要素保障；坚持领导联系重点项目全覆盖，健全快速反应协调机制，及时解决重点项目推进中的突出问题。

　　深入实施工业强市战略。落实供给侧结构性改革举措，实施工业“五大攻坚”，提升发展速度和质量效益，规模以上工业增加值增长9.1%。推动项目投资攻坚，抓好50个投资上亿元重大产业项目，确保10个重大项目竣工投产、20个重大项目加快推进、20个重大项目开工建设。推动创新驱动攻坚，出台深化科技体制改革政策，设立创新创业引导资金1000万元；加大技改投入力度，完成技改投资205亿元；实施20个技术创新项目，争创2个国、省企业技术中心，创建高新技术企业5户，新增1户企业上市。推动市场拓展攻坚，完善“天虎云商·资阳工业馆”“互联网+”平台，推动60户企业建立网上直销平台，组织100户企业“走出去”拓展市场，鼓励同等条件下优先采购本地产品，降低企业产品库存。推动平台建设攻坚，加快中韩创新创业产业化基地、空港经济区建设，强力推进国家级经济开发区创建工作，完善工业园区基础设施及功能配套，提升园区服务水平、创新活力及生产、就业、纳税能力。推动企业帮扶攻坚，出台支持实体经济政策，规范涉企收费行为，降低物流、融资费用，扩大直购电、富余电量消纳政策覆盖范围，推进天然气转供变直供；用好产业发展资金、应急转贷金，鼓励银行创新信贷产品，降低利率定价，支持有市场、讲诚信的企业转型升级、做实做强，大力推进企业在银行间市场发行债券，支持企业上市，破解企业融资难题；持续开展“走企业、解难题”活动，推动困难企业扭亏增效、停产半停产企业恢复生产。

　　加快发展现代服务业。实施现代服务业发展“五大行动”，推动服务业发展提速、比重提高、水平提升，增加值增长9.4%，社会消费品零售总额增长11%。实施产业提升行动，大力发展电子商务、现代物流、养老健康等先导型服务业，支持跨境电子商务发展；提质转型发展商贸流通、房地产等传统服务业，规划建设一批特色商业街区和重点商贸镇。实施企业培育行动，鼓励企业创建服务品牌和实施标准化管理，支持唯品会等龙头企业做大做强，力争新增服务业限额以上企业20户。实施平台支撑行动，利用简阳西部电子商务集聚发展区平台，加强电子商务、现代物流大企业招引集聚，启动建设安岳现代物流园区、乐至童家物流园区，推进城东物流园区建设。实施项目带动行动，力争安博物流等10个重点产业项目开工建设，确保万达广场一期等10个重点产业项目竣工投用。实施改革创新行动，启动首批国家级旅游业改革创新先行区试点、全省跨境电子商务先行先试工作，深入推进简阳、安岳国家级电子商务进农村综合改革试点和雁江区中心城区转型发展省级试点工作。

　　大力发展现代农业。突出“五大抓手”，推进农业标准化生产、规模化经营、品牌化建设、电商化销售，确保农民增收、农业增效，增加值增长3.2%。抓新型主体培育，支持龙头企业优化重组，推动企业、专业合作社、家庭农场规范化建设，完善利益联结机制，助农增收。抓特色优势引领，推进“八带九区十基地”建设，优化现代农业布局，积极发展新产业新业态，推动一二三产业融合发展；坚持以生猪为主导、山羊为重点、小家禽和水产为特色，推进“一村一品”和农业（畜牧、林业）重点县建设，突出抓好种养循环园区（基地）建设。抓农产品品牌建设，推行农业标准化生产，强化地方优质品种选育、保护和产品开发，加强“三品一标”认证，打造农业区域品牌，提升资阳农产品品牌影响力。抓幸福美丽新村建设，统筹推进扶贫新村和幸福美丽新村建设，完成安岳、乐至省级新农村示范县建设，建成幸福美丽新村350个以上。抓农业农村综合改革，引导农村土地经营权有序流转，培育多元经营主体；开展农村产权抵押融资、农村集体资产股份制改革等试点工作。抓好第三次全国农业普查工作。

　　着力发展县域经济。全力支持县域经济加快发展、竞相发展、追赶发展，支撑全市经济持续健康发展。做强特色经济，坚持工业强县、特色立县、富民强县，支持各县（市、区）根据区位条件、产业基础、资源优势，因地制宜发展优势产业，调优产业结构，培育特色经济。加强城镇建设管理，加快新区建设和旧城改造，完善功能、提升品质；抓好城镇路、水、电、气及污水、垃圾处理等设施建设。按照全省“洁净美好家园、建设宜居四川”行动要求，将中心城区市容市貌十大专项治理行动延伸到县城和乡镇，切实改善城乡环境。持续改善民生，丰富完善教育、卫生、体育、文化等资源和设施，提高质量和水平，促进公共服务均等化；在群众最急需领域办好一批民生实事，确保完成年度脱贫任务。加强投资促进，着力引进一批世界500强、国内知名企业及高附加值项目，引导在外资阳籍企业家返乡发展。

　　二、突出抓建设强管理，提升中心城区品质

　　扎实推进中心城区“改造完善提升攻坚年”行动，力争用三年时间，实现城市品质明显提升；按照“畅洁绿美、规范有序”要求，确保市容市貌实现显著变化。

　　提升城市规划水平。结合“六大规划”，按照雁江、简阳相向发展和成都、资阳一体化发展要求，研究航空都市发展方向、空间布局、城镇规模和形态。完成资阳城市总体规划修编，推进城市绿地、建筑风貌、保留山体等规划编制，做好主街主路、沱江九曲河沿岸城市设计并组织实施。

　　提升城市建设水平。扎实抓好“五大工程”建设，大力推进130个重点项目，完成投资97.6亿元。实施路网畅通工程，推进沱西滨江路、城东滨江路等8个续建项目，开工现代大道北延线、建设南路西延线等13个项目，启动二环路北段及沱四桥等9个项目前期工作，打通公园路延伸段等4条断头路，综合整治雁北路，畅通三区联系。实施干道提升工程，按照“七位一体”要求，重点抓好娇子大道、希望大道等4条干道和成渝高速资阳站入口提质升级，加快推进建设东路等12条道路“黑化”。实施园林绿化工程，启动国家级园林城市创建和凤岭公园、沱西滨江带改造建设，加快推进字库山公园二期、城东新区滨江路堤景观带等项目建设，完善提升主要街道重要节点的绿化和生态休闲公园建设，加快城区保留山体绿化。实施公共配套工程，推进能源、通信、供排水等多网同建，新改建一批污水排放、公厕等环卫设施，开工建设垃圾发电项目，加强路灯照明等建设管理，加快推进农副产品交易中心、孙家坝铁路市场建设，规划建设城区停车场和汽车充电桩。实施民生改善工程，加快推进兰家坡、朝阳花园等9个棚户区改造，启动垃圾填埋场片区等7个城中村改造项目，新建茶花苑等3个农贸市场，实施56条小街小巷整治。

提升城市管理水平。持续推进十大专项治理行动，巩固扩大治理成果，实现治理全覆盖。完善城市管理体制，推进市区城市管理执法体制改革，组建城市行政执法局。建立完善工作机制，纵向健全“市、区、街道、社区”四级城市治理体系，横向完善市级部门主动参与、联动执法的大城管格局；加强宣传动员，引导市民自觉参与城市管理，形成良好的城市管理氛围。推进城市管理科学化，制定城市管理规范标准，将管理标准精细到每条道路，实现标准化管理全覆盖；拓展数字城管平台，提高城市管理信息化水平。

　　三、持续打基础优环境，改善生产生活条件

　　坚持基础先行，完善基础设施网络体系，提升保障发展的能力和水平。

　　加快交通网络建设。推进以机场为核心，高速公路、快速通道为重点的综合交通建设，完成投资120亿元。抓大通道建设，配合推进成都天府国际机场全面开工建设；启动资潼广高速、成都经济区环线东段建设，全力争取成安渝高速公路、成都经济区环线高速南段主线工程竣工，加快推进机场高速南段、乐三高速前期工作。抓中通道建设，扎实推进资三、简三快速通道前期工作，确保国道318线红日大桥建成通车。抓小微通道和站点建设，启动资阳城北汽车客运站迁建前期工作，推动雁江、简阳城市公交与成渝客专快速对接，新改建农村公路530公里。

　　推进水务设施建设。统筹推进城乡水务设施建设，完成投资33亿元。突出抓好水利工程建设，推进毗河供水一期工程，完成投资15亿元，力争完成二期工程可研编制；推进关刀桥水库、东风渠六期续建配套工程及中小微型水利工程建设，解决10万农村人口饮水安全问题。全力保障中心城区供水安全，强力整治老鹰水库周边环境，启动净水入库工程建设，切实改善水质；建设老鹰水库至城区第三期饮水管道；启动城区备用水源、城东水厂建设；完善城市供水管网，启动城区输水管道扩容增压工程，保证饮用水的安全性可靠性。加强城市排水设施建设，建设和完善九曲河、沱江过江管道等城市截污干管及配套管网，提高生活污水、工业废水集中收集处理率，充分发挥污水处理厂工程效益。

　　加强能源通信设施建设。抓好能源设施建设，完善220千伏双环网，推进农网升级改造，完成投资14亿元。进一步抓好安岳油气田勘探开发，力争启动天然气脱硫厂建设。抓好通信设施建设，启动中国移动川南数据枢纽中心建设，推进4G网络、乡镇光纤全覆盖，农村光纤覆盖面达50%以上。

　　加快推进天府新区资阳片区建设。加快营造天府新区资阳片区旅游氛围，全力推进长岛国际会议中心、滨湖公共空间、世茂产业生态城等3个项目形成接待能力。

　　抓好生态文明建设。加强环境保护，大力整治大气、水体、畜禽养殖污染，逐步取缔沱江及水库肥水养鱼，加大农村面源污染治理，全面完成总量减排目标任务；严把建设项目准入关，全面清理违法违规建设项目，严厉打击环境违法行为；深入推进城乡环境综合治理，改善城乡环境面貌。加强生态建设，大力推进生态县（市）、生态镇、生态村建设，实施天然林保护、退耕还林、植树造林工程，新营造林15万亩，新增森林面积3万亩。加强耕地保护，抓好12.8万亩高标准农田建设，推进永久基本农田划定，实现耕地占补平衡。

　　四、深入抓改革扩开放，增强发展动力活力

　　着力推进各项改革，全面扩大开放合作，充分激发发展活力和动力。

　　实施改革攻坚。深化行政审批制度改革，加大简政放权力度，转变政府职能，建立健全权力清单、责任清单、负面清单，实施行政审批“三个集装箱”制度。加快推进投融资体制改革，推广PPP模式，采取基金、企业债券等多种形式融资；推进金融改革创新，完成资阳农村商业银行组建，推进安岳、乐至农村信用联社改制为农村商业银行，支持民生村镇银行增资扩股。深入推进财税体制改革，实施“营改增”和国地税征管制度改革；深化公共资源交易改革；加大政府购买公共服务力度；积极稳妥推进公务用车制度改革。深化医疗卫生体制改革，全面启动城市公立医院改革。统筹推进教育、文化、价格等改革。

　　扩大对外开放。大力招商引资，借力西博会等重大平台，自主举办基础设施建设等专题招商活动，精准对接“三类500强企业”，推动招大引强、引资引智，到位国内省外资金410亿元，引进外资0.6亿美元。深化开放合作，全面对接“一带一路”、长江经济带等国家战略，增进与沿线国家和地区的交流合作；以成渝客专建成通车为契机，加强与成都、重庆的深度合作；与美国纽波特纽斯市建立友好城市；推进厅市、校市等战略合作取得实效。扩大对外贸易，开展资阳企业丝路行等活动，实现进出口2.1亿美元；积极参与四川内陆自贸区争创工作。

　　五、着力补短板惠民生，提高群众生活水平

　　持续推进社会事业发展系列方案和行动计划，加快实施一批惠及民生的重大工作和重大项目，确保取得明显成效。

　　持续改善社会民生。扎实推进民生实事，整合各类财政资金，优先保障民生支出，完成省下民生工程和民生大事，办好市委、市政府确定的16件民生实事。健全社会保障体系，实现社会保险法定人群全覆盖，推进“五险合一”统一征收，完成城乡医保一体化改革；启动雁江区殡仪馆迁建等项目，力争市民政福利园投用；全面开展城乡低保对象普查和精准识别工作，提升救助水平；统筹发展城乡养老服务。全力推进脱贫攻坚，落实精准扶贫、精准脱贫要求，继续实施扶贫开发整村推进，加大干部驻村帮扶力度，确保113个贫困村、6.7万贫困人口全部摘帽脱贫。加强就业创业工作，全面落实大众创业、万众创新政策，促进农民企业家和农民工返乡创业、高校毕业生自主创业，带动和扩大就业，实现城镇新增就业2.25万人；建成创新创业孵化器。加快保障住房建设，实施棚户区改造5787户，建成保障性住房860套，抓好农村危旧房改造。

　　大力发展社会事业。推动教育事业发展，实施新一轮学前教育三年行动计划，再开工建设18所公办幼儿园，加强城镇小区配套幼儿园规划建设管理；实施中小学大班额解决方案，加快96所中小学校建设改造，实施“信息化建设两年攻坚行动”，促进义务教育均衡发展；加快推进成都体育学院迁建工作；加强师德师风建设，培养一批好校长、好老师。提升医疗卫生服务水平，实施医疗卫生服务能力提升三年行动计划，新建市人民医院业务大楼等4个项目，建成3个省级重点专科，与华西医院和省人民医院合作，全面提升医技水平和服务质量。加强公共卫生服务体系建设，强化重大疾病防控和预警预报，广泛开展爱国卫生运动。大力发展文化事业，做好文化艺术中心、博物馆等项目前期工作，加快文化阵地建设；重视文化展示传承，培养文艺人才队伍，办好资阳文化艺术节等特色文化活动，打造特色精品文化。加快完善体育设施，启动市全民健身中心建设，加强城区公园、住宅小区体育设施建设，实施城区保留山体上山步行道建设，打造群众就近健身锻炼场地。优化人口计生服务，全面落实两孩政策。全面启动资阳市志编修。加强外事侨台、统计、档案、民族宗教、红十字、保密、老龄、残疾人、妇女儿童等工作。

六、持续解难题防风险，维护社会和谐稳定

　　密切关注经济下行可能引发的矛盾和问题，提升防范风险、维护稳定的能力。

　　防控经济风险。抓好企业风险防控，着力防止企业资金断链、企业大规模停产倒闭和职工大量下岗失业。抓好金融风险防控，巩固金融生态环境建设成果，推进信用资阳建设，加强投融资监管，坚决遏制非法集资、高利放贷，打击恶意逃废银行债务行为，严密监控网络借贷、股权众筹等新型金融业态，防范滋生新的风险。抓好房地产风险防控，加大稳定住房消费政策支持力度，继续办好房交会，推进房屋征收、棚户区改造货币化安置，推动房地产去库存，稳妥推进问题楼盘处置工作，促进房地产市场良性发展。继续加强政府性债务风险防控，规范政府举债行为，用好国家债务置换政策，积极化解存量债务，保持政府债务在限定额度之内。抓好建设领域遗留问题化解，全面落实毛地拍卖遗留问题处置方案，逐步化解毛地拍卖遗留问题；持续推进安置房三年攻坚行动，今年竣工227万平方米。

　　创新社会治理。落实依法治市要求，提升社会治理水平。加强司法行政工作，深入开展“法律七进”活动，启动“七五”普法工作，提高全民法治意识，加强法律援助、社区矫正和人民调解工作。加强基层社会治理，推进社区公共服务综合信息平台建设，做好第十届村居民委员会换届选举工作。加强信访维稳工作，依法规范信访秩序，完善领导包案、下访机制，着力化解一批城市建设领域、民间借贷纠纷、涉法涉诉等信访积案难案。加强社会治安综合治理，深化“平安资阳”和社会治安防控体系建设，严厉打击违法犯罪活动，增强群众安全感。加强国防双拥工作，抓好国防动员和后备力量建设，推动和保障军民融合深度发展，加强全民国防教育，进一步强化国家安全、保密和人民防空工作，启动新一轮全国全省双拥模范城创建。加强安全和应急管理工作，深化依法治安，强化安全生产责任落实，健全安全隐患排查治理体系，推进雁江、简阳省级食品安全示范县创建工作，提升突发事件预防和处置能力，坚决遏制重特大安全事故发生。

　　七、坚持改作风提效能，建设人民满意政府

　　坚决服从市委领导，自觉接受人大、政协及社会各界监督，按照“三严三实”和“为民务实清廉”要求，持续加强政府自身建设，提高政府工作水平。坚持依法行政，坚持政府常务会会前学法制度，强化法治思维，运用法治方式推动发展、维护稳定；认真办理人大代表建议和政协提案。规范政府运行，完善规范性文件和重大行政决策合法性审查制度，推动科学民主依法决策；坚持守规矩、讲纪律，规范行政行为，确保令行禁止、政令畅通；加强政府绩效管理和政务督查，确保政府高效、有序、协调运转。改进工作作风，保持奋发有为的精神状态，敢于担当、勇于创新、狠抓落实，切实解决工作效率和质量不高、不按规则办事等问题；坚持群众工作路线，深入开展“走基层、解难题”，帮助群众办实事办好事。加强廉政建设，认真落实党风廉政建设责任，持续深化正风肃纪专项行动，切实解决发生在群众身边的“四风”和腐败问题。抓好中央“八项规定”、省市“十项规定”精神落实情况的监督检查；加强监察审计工作，严肃查处违法违纪行为，建设廉洁政府。

　　各位代表！站在“十三五”发展新起点，我们深感使命光荣、责任重大。让我们在市委的坚强领导下，在市人大、市政协的监督支持下，同心同德、拼搏进取，努力实现“十三五”良好开局，为加快建设“三区一都市”，奋力打造全省经济强市，实现与全国全省同步全面建成小康社会而努力奋斗！
